# Recurrent arteriovenous graft thrombosis in COVID‐19 positive patient

**DOI:** 10.1002/ccr3.4732

**Published:** 2021-08-30

**Authors:** Anil Kumar Singh, Saurabh Bansal, Namrata Singhania, Girish Singhania

**Affiliations:** ^1^ Department of Internal Medicine Geisinger Community Medical Center Scranton Pennsylvania USA; ^2^ Department of Internal Medicine University of Illinois College of medicine at Peoria Peoria Illinois USA; ^3^ Department of Hospital Medicine Mount Carmel East Hospital Columbus Ohio USA; ^4^ Department of Nephrology University of Utah Salt Lake City Utah USA

**Keywords:** arteriovenous graft thrombosis, COVID‐19‐associated coagulopathy, end‐stage renal disease

## Abstract

Hypercoagulability is one of the common complications seen in COVID‐19. It can lead to multiple thromboembolic complications. Recurrent arteriovenous graft thrombosis can be one of complications from this pathophysiology.

## Graphical abstract

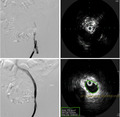

## 1 INTRODUCTION

Coronavirus disease 2019 (COVID‐19) has been tied to many complications such as hypercoagulability. It is more commonly seen in patients admitted to intensive care unit. Herein, we report a case of an end‐stage renal disease patient who developed recurrent arteriovenous graft thrombosis in the setting of COVID‐19.

Coronaviruses are RNA viruses with viral spike (S) which binds to host angiotensin‐converting enzyme 2.^1^ It spreads via respiratory droplets between close contacts. The virus may remain infectious in aerosols for hours and on surfaces up to days. Infection may be asymptomatic or may result in an acute respiratory disease with fever, shortness of breath, and cough. Bilateral pneumonia, acute respiratory distress syndrome, or death may occur.^1^ Some patients have experienced gastrointestinal symptoms such as diarrhea or other atypical symptoms.^2^ Hypertension, heart disease, and chronic lung disease are risk factors for severe disease. The coronavirus disease 2019 (COVID‐19) has brought many unique pathologies such as hypercoagulability which can cause various thromboembolic complications, especially in critically ill patients.^3^ Herein, we present a case of recurrent arteriovenous (AV) graft thrombosis in the hemodialysis patient diagnosed with COVID‐19.

## 2 CASE DESCRIPTION

**FIGURE 1:**
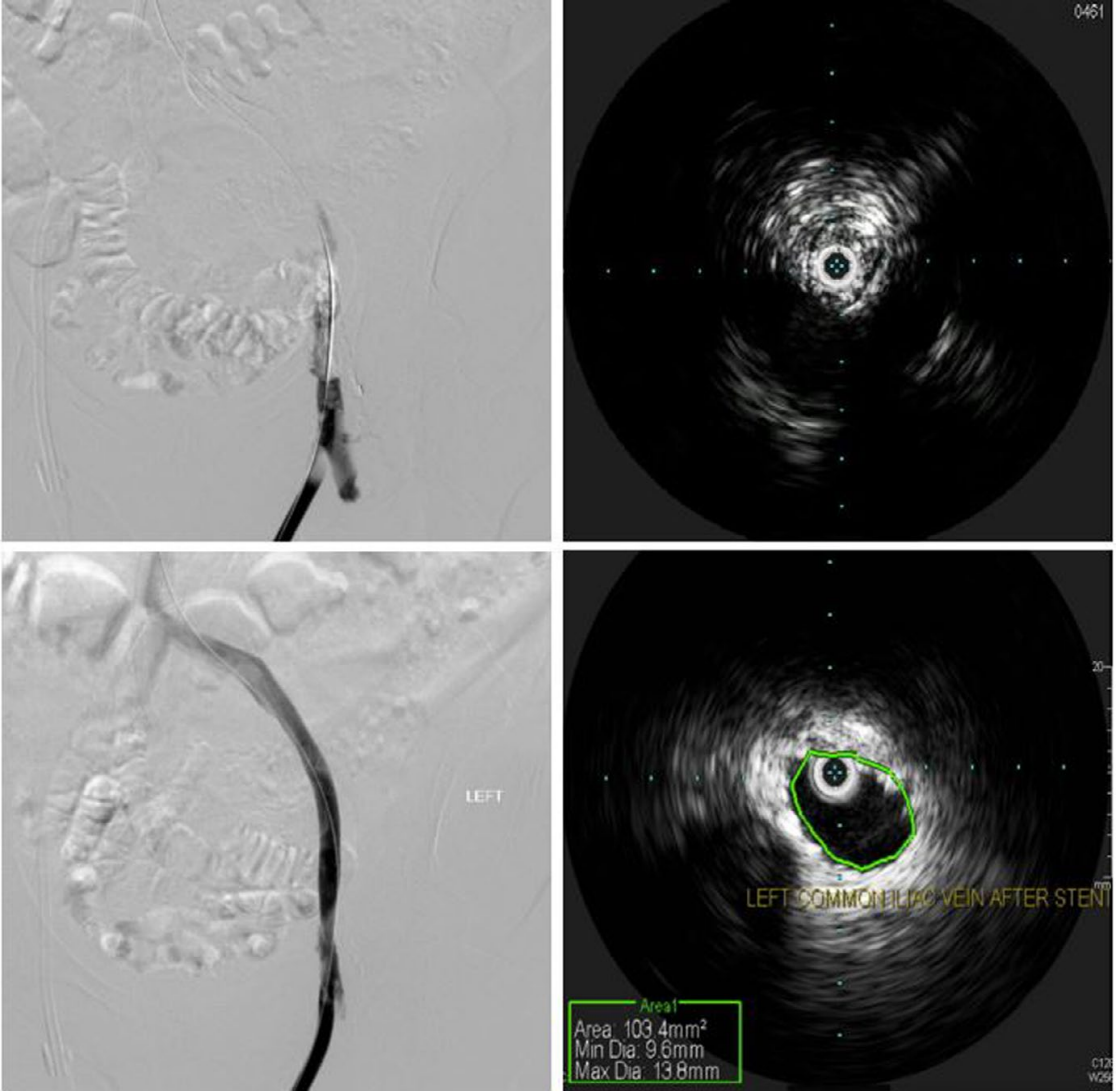
(A) Angiography of lower extremity AV graft showing occlusion of the graft; (B) IVUS showing thrombus occluding the left common iliac vein; (C) Angiography of lower extremity AV graft after thrombectomy with successful blood flow; (D) IVUS showing successful revascularization of left common iliac vein after thrombectomy and stent. AV, arteriovenous; IVUS, intravascular ultrasound

An 84‐year‐old African American female with past medical history of end‐stage renal disease (ESRD) on hemodialysis through a left lower extremity arteriovenous graft, type 2 diabetes mellitus, and atrial fibrillation on warfarin came to the emergency department after she was found to have temperature of 101°F at her dialysis center. She was complaining of mild nonproductive cough but denied any shortness of breath or any sick contacts. In emergency department, her temperature was 99.1°F, blood pressure 146/77 mmHg, heart rate 73 beats/min, respiratory rate 17/min, and oxygen saturation 98% on room air. She was diagnosed with COVID‐19 detected by polymerase chain reaction (PCR) and eventually discharged home. She came back again 1 week later with shortness of breath and was admitted due to hypoxemia. Her physical examination was unremarkable other than crackles in lungs bilaterally. Her electrolytes were normal. Her INR was sub‐therapeutic at 1.4. Her platelet count was 114,000/mm^3^, prothrombin time (PT) 17.8 s (reference range [RR]: 11.9–14.7 s), activated partial thromboplastin time (aPTT) 38.4 s (RR: 23.3–35.3 s), and D‐dimer 3.28 mcg/ml (RR: <0.4 mcg/ml). Her severe acute respiratory syndrome coronavirus 2 (SARS‐CoV‐2) PCR was again positive. She had elevated venous pressures during hemodialysis and hence doppler was ordered which showed homogenous echoes suggestive of AV graft thrombosis. She was started on heparin drip (bolus at 70 units/kg and drip 15 units/kg/h). Angiography and intravascular ultrasound were performed which showed thrombosed AV graft (Figure 1A,B). Thrombectomy and stent placement were performed successfully with good flow post‐procedure (Figure 1C,D). She had uneventful hemodialysis afterward and was discharged. A day after discharge, she came back again with diarrhea. Her repeat SARS‐CoV‐2 PCR was still positive. Her INR was 1.9. She was again found to have high venous pressures during hemodialysis and hence doppler was repeated which was positive for recurrent thrombosis of AV graft. She was started on heparin drip at the same previous rate and vascular surgery decided to place a tunneled dialysis catheter and hold thrombectomy till her SARS‐CoV‐2 PCR turns negative, due to concerns of hypercoagulability in the setting of COVID‐19.

## 3 DISCUSSION

Novel COVID‐19 first originated from Wuhan, China and has spread rapidly to all over the world.^1^ It spreads via respiratory droplets between close contacts and patients usually present with respiratory symptoms although atypical symptoms like altered mental status in elderly patients have been reported.^2^ There have been increased evidence of hypercoagulability seen in patients infected with COVID‐19. Venous thromboembolism (VTE) is common in acutely ill patients with COVID‐19 and has been termed as COVID‐19‐associated coagulopathy (CAC).^3^ An autopsy study showed as high as 58% incidence of VTE.^4^ The pathogenesis of CAC is incompletely understood. All three categories of “Virchow's triad” appears to be implicated including endothelial injury as evident by direct invasion of endothelial cells by the virus along with increased cytokines and complements, stasis due to immobilization in hospitalized patients, and hypercoagulable state due to changes in circulating prothrombotic factors such as D‐dimer and fibrinogen.^5^ Risk factors for CAC are males with obesity and other chronic medical comorbidities, especially cardiovascular disease, hypertension, diabetes mellitus, and ESRD.^4^ Common laboratory findings include high D‐dimer and fibrinogen, mildly prolonged PT and aPTT, and mild thrombocytopenia.^4^ Elevated D‐dimer levels appear to correlate with illness severity as well as increased mortality.

COVID‐19‐associated coagulopathy appears to clinically behave differently from disseminated intravascular coagulation (DIC).^3^ The major clinical finding in CAC is thrombosis and high fibrinogen, whereas acute DIC often presents with bleeding and low fibrinogen.^3^ Management can be challenging due to absence of high‐quality data. Whether therapeutic dose anticoagulation (heparin 5000 IU subcutaneous every 8 h or enoxaparin 40 mg subcutaneous once a day) should be offered to everyone with COVID‐19 remains unclear. Some authors have suggested using intermediate dose (60 mg subcutaneous once a day) of low molecular weight heparin in patients with significantly elevated d‐dimer levels due to the high percentage of patients with VTE despite receiving prophylactic anticoagulation.^6^, ^7^ Full‐dose anticoagulation (heparin bolus and drip as in our patient or enoxaparin 1 mg/kg subcutaneous twice a day) is recommended for individuals with documented VTE or with recurrent clotting of intravascular access devices unless contraindicated.

## 4 CONCLUSIONS

COVID‐19‐associated coagulopathy, Sub‐therapeutic INR, history of end‐stage renal disease, and diabetes mellitus placed our patient at higher risk for recurrent AV fistula thrombosis.

## CONFLICT OF INTEREST

None declared.

## AUTHOR CONTRIBUTIONS

AKS and NS wrote the manuscript and reviewed the literature. SB and GS revised the manuscript and reviewed the literature.

## ETHICAL APPROVAL

Ethic committee was not consulted for approval as it is a case report and all possible efforts were made to maintain complete anonymity.

## CONSENT STATEMENT

Published with written consent of the patient.

## Data Availability

Data sharing not applicable – no new data generated, or the article describes entirely theoretical research.
